# A Layman’s View of Tuberculosis

**Published:** 1905-05

**Authors:** Eli P. Smith

**Affiliations:** Associate Editor of the *Birmingham Daily News*


### A LAYMAN'S VIEW OF TUBERCULOSIS.*

BY ELI P. SMITH,

Associate Editor of the *Birmingham Daily News*.

Mr. President and Gentlemen of the Alabama State Medical Association:

I would consider myself utterly devoid of both gratitude and feeling were I to fail to keenly appreciate the honor which your distinguished president has conferred upon me by inviting me to appear before this learned body to express to you my views—the views of a layman who has had actual experience—of the practical side of tuberculosis.

It is no exaggeration to say that no other single disease is to-day commanding a wider range of study or more thoughtful consideration than consumption. This is not only true as to the members of the medical profession, but it applies as well to the intelligent lay mind. All are uniting in an earnest endeavor to devise some means to stay the ravages of the great "white plague," and therefore it follows that every particle of information worthy of consideration bearing upon the subject is being eagerly sought by thoughtful men everywhere and carefully weighed, with the single purpose of solving the mighty problem at hand.

Nor is this intense interest in the subject surprising when we consider for a moment the awful destruction which is being wrought by this malady year by year. According to the government reports the number of deaths in the United States from consumption during the census year of 1900 was 111,059, or 10,688 per 100,000 from all causes. In other words, one out of every ten persons who die from disease in this country is a victim of tuberculosis. No other disease in the entire category claims so large a proportion of the human family, not even pneumonia, though the latter is close behind the leading destroyer. It is this fearful record which makes the consideration of tuberculosis of intense personal concern to so large a percentage of our population. To employ a commonplace phrase, the question is brought

*Read before Alabama State Medical Association.

directly home to them. Viewed in this light it is easy to comprehend the vital interest which the masses of the intelligent population must feel in this subject, the eagerness with which every popular publication relating to it is read, and the readiness with which every new remedy suggested is tried.

FROM SCHOOL OF EXPERIENCE.

In my remarks on the practical side of tuberculosis I shall not attempt to employ technical or scientific phraseology. I am not a man of medicine. I have never in all my life seen the inside of a medical book. I have read nothing on this very subject which I am discussing, except a number of papers written by my physician, Dr. S. G. Bonney, of Denver, Colo., and read by him before the American Medical Association, and such newspaper and magazine publications relating to the treatment of consumptives by modern methods as have appeared during the past three and a half years. What I shall tell you, therefore, will be chiefly from practical and hard experience, coupled with close observation taken, both in my own case and in the cases of hundreds of other "lungers" with whom I came in contact while battling with the disease in the dry climates and high altitudes of the far West—the battlefield where so many desperate and determined fights are constantly being waged with this cruel destroyer and where, through the kind assistance of nature's own remedies, reinforced by wise medical counsel and common sense and a firm will-power on the part of the patient, scores and hundreds of valuable lives are being yearly saved from an untimely grave.

I am frank to say that before I had myself been attacked by tuberculosis I had no adequate conception of what care of myself meant. I did not halt sufficiently long in the mad rush incident to modern business-life to weigh the meaning of moderation in effort, the necessity of keeping up one's vitality, the value of fresh air, or the need of hygienic precautions and safeguards. I taxed my physical being to the full limit, and realized my blunder only when, at the end of a day by terrible effort, October 19th, 1901, I utterly collapsed and bespattered my office from one end to the other with blood, emerging from the ordeal so weak and depleted that I was with difficulty gotten to my home in a carriage. I had worked for thirteen years on an average of fourteen or fifteen hours daily, and during that entire period had lost but thirty days on all accounts, including both vacation and idleness. I had kept myself bent over a desk, paying no heed to the ventilation of either my office or bedroom, and giving but little, if any, more attention to my diet and general manner of life.

Twelve years prior to my first series of hemorrhages I had suffered a severe attack of pneumonia, and hope for my recovery had at one time been practically abandoned. The attack followed a spell of measles, and my recovery was considered little less than miraculous, but I had employed my saving force—will-power—and with its aid came out alive. This pneumonia attack left my lungs in a weakened condition, a fact which I knew, but the importance of which did not then impress me. No member of my family, so far as I know, had ever suffered from tuberculosis, so that even if we should subscribe to the now exploded theory of hereditary consumption it would not apply in my case.

TUBERCULOSIS REALIZED.

But to deal with my attack of tuberculosis in detail. As soon as I came to a realization of the fact that I had suffered from a series of hemorrhages of the lungs I readily understood that I was a victim of tuberculosis. My Birmingham physician never told me so. He must have known, but he doubtless preferred to adopt the tactics which in my judgment too many doctors employ—the policy of not frankly and promptly advising the tubercular victim at the very beginning of his trouble the exact nature of his ailment for fear of unduly alarming him.

Right here I desire to express my unqualified disapproval of this policy—a policy which I am pleased to say is rapidly disappearing. I am firm in the conviction that it is the sacred duty of the doctor to promptly inform a consumptive at the outset of his trouble and seek at once to impress upon him the importance of early and strenuous action on his part to abate the disease. I regard every knowledge of the presence of the disease as a prerequisite to a winning fight, provided the victim will accept the statement of the physician as a fact and make up his own mind to spare no effort on his own part to assist nature in throwing off the infection. Of course, right here many doctors encounter a serious difficulty, which lies in the fact that very many consumptives flatly refuse to believe that they have tuberculosis. This refusal springs out of the old fiction that to accept the idea that you have consumption is tantamount to having your death warrant read to you. This should not and will not be the case, provided the patient will upon receiving first knowledge of his actual condition accept it as the truth and set about to do battle with the disease according to modern common-sense methods, accepting the situation as he finds it and making the best of it, always bringing to bear every iota of will-power at his command.

My own experience has convinced me that nature never intended that man should be otherwise than healthy. In other words I firmly adhere to the theory that it is unnatural to have consumption or any other disease. But after having contracted disease the entire tendency of nature in my opinion is to expel it from the body. This theory being accepted, the part which the physician and the patient himself must play is to assist nature in accomplishing the work of expulsion.

When during my illness I grasped this theory and determined to do my part in aiding nature I found that my progress towards recovery was most satisfactory and rapid. To sum up this point, I believe that early acceptance of the truth that one is a victim of tuberculosis and a strong and unshaken determination to whip the fight, followed by the immediate inauguration of unflagging effort to that end, is about seventy-five per cent. of a successful battle with this disease.

### TREATMENT IN COLORADO.

But to continue the discussion of my own case. It was one month after my first series of hemorrhages before I was able to travel. Then I went to Denver and placed myself under the treatment of Dr. S. G. Bonney, whom I very naturally regard as the greatest lung doctor in America. I would be a base ingrate if I placed a smaller estimate upon the ability of this man, who guided my footsteps into the paths which led to a restoration of my shattered health.

Up to that time no medical authority had told me that I had tuberculosis. My home doctor had confided to me the information that I desired to travel in the West. I asked him how he had ascertained my desires in this matter, but he only smiled. When I left home it was much like shooting into the darkness. I had no fixed idea of where I expected to find a temporary habitation or how long I would be absent, but I harbored an unshaken determination to get well, despite the misgivings which I could clearly see depicted upon the faces of friends who bade me good-by. However, I was fortunate enough to gain the company of a friend, who was suffering just as I was, having been attacked while a student at Bellevue Hospital in New York. This friend had been advised by eminent medical authorities in New York to go to Denver. He had been equipped with letters of introduction by them to lung specialists in that city, and I promptly joined him.

Arriving in Denver we had the good luck to meet another friend who was under treatment of Dr. Bonney and secured board at the same house where he was stopping. He described to us in glowing terms the manner of treatment and recited the wonderful physical progress he had made. At the boarding-house were fourteen "lungers," the happiest and jolliest aggregation of health seekers I have ever seen. Their optimism immediately became infectious, and we joined the party at once, even before we had sought medical advice, adopted their method of "chasing the cure," as it is very appropriately termed.

I few days later when I found myself closeted in the private office of Dr. Bonney for an examination and had been told by him that I too was a "lunger," but that I had acted wisely in coming early to a climate where the air is pure and the largest amount of sunshine is to be had. I heaved a sigh of relief. I felt relieved because he frankly detailed to me my exact condition, and told me almost to the day the period of time which, in his judgment, would be required for me to recover sufficiently to enable me to return home and live in Alabama, provided, of course, I pursued here in a large degree the same manner of life which he was about to teach me in Colorado. I accepted the doctor's statement implicitly. I believed every word he said and subsequent developments have proven that my confidence was not in the slightest degree misplaced.

Right here I desire to add that this feature of the case has very much to do with assisting a consumptive along the road to recovery—faith in his physician and scrupulous adherence to his mandates.

During the first three months of my stay in Colorado I played the role of the fattening pig. That is to say, I was taught that what I must first have was flesh, and I bent my every energy to gaining it. On my arrival I weighed 132 pounds. My normal weight had been 142 pounds. In fact the latter figures represent my maximum weight before I contracted tuberculosis. Today my weight ranges from 168 to 175 pounds, my endeavor being not to allow it to drop below the former sum.

### FRESH AIR AND SUNSHINE.

In the beginning of my treatment four things were forcibly and indelibly impressed upon my mind—the need of flesh, the necessity of plentiful quantities of fresh air, rest of body and mind, and moderation in exercise. I was told that if I indulged in a large amount of exercise I would work off my surplus flesh as rapidly as I put it on, that if I worked I could not gain flesh and that my impaired lungs required all the wholesome fresh air and sunshine possible for them to obtain.

For three months myself and the other members of our little colony spent each day sitting in the genial sunshine—winter sunshine—in the back yard of our boarding-house. When the weather became cold we would cuddle up in a large piano box open on one side or else nestle close to the protecting side of the high board fence, but always where the sun's rays could reach us. When snow covered the ground we wrapped up in overcoats and rugs and braved the elements. At night I kept four windows wide open in my bedroom, a practice which I still pursue.

I recall that during one extremely cold spell I slept with my four windows open during a night when the thermometer dropped to twenty-one degrees below zero, but I was always careful to see that I had adequate covering. I soon found that by always breathing fresh air and by frequent resort to cold baths I became impervious to colds and it is a fact that I have never taken cold since I contracted tuberculosis three and a half years ago.

While I was strenuously pursuing the manner of life described above I gained flesh steadily. Happily I had an "iron clad" stomach and was able to digest large quantities of nourishing food. A glass of cream at each meal, raw eggs, a tablespoonful of emulsion of mixed fats in hot water twice a day, small pellets of strychnine, now and then a course of Fellows' hypophosphite, a little digitalis when my heart seemed to require it, were all adjuncts to my regular diet of roast beef, steaks, eggs, butter, chops and like wholesome and nourishing food. During all this treatment I made it a rule to allow nothing to worry me. I kept my mind diverted by reading, writing on a small table in the open air and swapping yarns with my companions, so that the time passed away pleasantly as well as profitably.

A TRIP TO CALIFORNIA.

At the end of three months of this treatment I had made such a substantial physical gain that my Denver doctor consented for me to do some of the traveling and sight-seeing which my home physician had insisted that I was so anxious to indulge in. I made short side-trips out from Denver, but in April became sufficiently bold and strong as to go to California where I spent three weeks. When summer came I "trailed" to a delightful resort far back in the Rocky Mountains at an elevation of almost 8,000 feet above the sea where I joined another jolly colony of "lungers" and we spent three months twenty-five miles from the nearest town or railroad. Our life was in the open air all the while, free and easy, and in every way conducive to completing the cure which I had begun in Denver.

When I returned home in September eleven months after my first series of hemorrhages I was weighing 166 pounds and had never felt better in my life. I had been discharged as "conditionally well," which meant that I would be running no special risk in resuming my newspaper work, provided I was moderate in all things and always kept up my vitality while at the same time seeing to it that my lungs were constantly and continually fed upon fresh air. However, my doctor had warned me that during my three months' stay at an extreme altitude I had become very full-blooded and upon returning to a much lower altitude I might suffer another series of hemorrhages if I did not exercise due caution. But I was so delighted to return to my work and friends, not in a "wooden overcoat," to use the picturesque language of the Western lunger, but in the rosy vigor of health, that I disobeyed this injunction. I plunged headlong into my work during a period of hot and depressing weather, and paid the penalty for my foolhardiness.

Within three days of the first anniversary of my previous hemorrhages I began bleeding again. As in the first instance the attack came while I was at my desk, and the manner in which I decorated the floor of my office with blood might have led an unsuspecting visitor to conclude that it was a slaughter-pen. These hemorrhages began at 1:30 o'clock one Thursday afternoon and continued at intervals until the following Saturday night, during which time I lost thirteen pounds in weight.

I was more provoked than alarmed, for at the very time when I was flat on my back heaving up blood, *The Birmingham News*, with which I am connected, came out with an article from my own pen, which had been prepared several days before my illness, telling the public how I had been cured of consumption and advising all sufferers to go and do likewise. While annoying in the extreme the humor of this situation could not but appeal to me. I communicated with my Denver doctor by wire and he advised me not to be uneasy, assuring me that my present attack was rather a readjustment to the lower altitude than otherwise, due to over-exertion during the warm weather. He termed it an "overflow," and judging from the amount of blood I lost, I am quite prepared to subscribe to the idea that it was not only an overflow, but a bloody flood. However, just two weeks from the day the attack came upon me I was back at work, and in four weeks more had regained all my lost flesh and have never from that day to this given the slightest evidence of a recurrence of these hemorrhages.

### ON GUARD FOR GERMS.

I attribute my present good health to the manner of life which I pursue, which while not as correct as it should be, is sufficiently proper for me to keep the "bugs" at arm's length, so to speak.

I realize fully that I have merely abated the disease and that if my vitality runs low I will be subject to recurrence of it. I know that on my left lung, which was the infected organ, there is an old scar liable to reopen at the hands of this insidious enemy the moment I am caught off guard. Therefore, I still follow as far as possible the manner of life which I adopted in the West. I can not live outdoors all the while and pursue my professional duties, so I do the next best thing by keeping my office well ventilated the year round, sleeping every night in the year in a room without heat and with four windows wide open, not even a curtain fluttering before them to obstruct the air currents; I endeavor to be moderate in my habits (though I must confess that I fall far short in this respect); and last but not least I eat only the most nourishing foods and these in plentiful quantities at regular hours.

When I find that my weight is receding, I put on more steam by taking on a side line of cream, raw eggs and emulsion of mixed fats. Last January my weight ran down to 162 pounds and I at once resorted to this plan, and in five weeks had carried my weight back to 170 pounds. Of course, my excellent digestion is a tremendous factor in aiding me to keep up my weight and general vitality. In the same connection I believe also that my will power and naturally optimistic temperament are important elements in my favor.

On one occasion my Denver physician told me that he had never in all his experience treated a patient who displayed greater natural power of resistance and he congratulated me upon possessing that characteristic, which I realize is very important to any person who is attacked by a wasting disease.

Still another feature of my present manner of life which must not be overlooked is the cold bath. It is my unvarying rule to take a cold plunge bath in a cold bathroom every morning in the year no matter what the state of the weather may be. This bath together with the system of ventilation which I employ both at my office and at home I feel sure is what renders me impervious to taking cold. A cold is an exceedingly dangerous companion for a consumptive and he should be overwatchful to avoid one. He can do so if he will not allow himself to become overheated and will always remain close to nature. But he should avoid overcrowded or overheated rooms and should not dress too heavily, rather adapting his dress to the climate and weather conditions. All of this can be easily conformed to if a little thought and effort are employed.

With regard to climatic influence in the treatment of tuberculosis, I regard a high altitude and extremely dry climate as important only in expediting the abatement of the disease and more readily insuring the cure. In my judgment these elements are not absolutely essential except in extreme cases. Consumption can be abated in Alabama if the patient will conform to the same manner of life and treatment here as I followed in Colorado, provided he begins in time. Here is the keynote to the entire proposition—an early beginning.

It is no exaggeration to say that thousands die every year who could have been saved had they realized their true condition early and adopted the open-air treatment. However, the trouble is that in very many and possibly a majority of cases the disease has gained great headway before the patient becomes fully aware of the fact, or if he suspects that he has it he is apt to fail to realize the importance of beginning the fight without delay until his vitality is at such a low ebb that he is scarcely able to travel to a more congenial climate.

### DO NOT DESIRE THE TRUTH.

The average man or woman does not desire to be told the truth about this disease, if the truth means that that man or woman is afflicted with it. As I have already stated this attitude grows out of the old fiction that consumption is incurable, and is undoubtedly responsible for the great caution which many physicians exercise in divulging to a consumptive the truth as to his real condition.

The solemn duty of the hour devolving alike upon the medical profession, the press and the intelligent layman is the education of the masses to the fact that consumption can be abated and that nature's own elements—God's sunshine, fresh air and wholesome food—furnish the cure. It has been amply demonstrated that these elements can abate tuberculosis in the most unfavorable climates.

The municipality of New York has proven it on Blackwell's Island where it maintains a consumptives' colony, the members of which pursue the same open-air treatment as is practiced in Colorado and New Mexico, and where more victims recover than die. The State of Massachusetts, whose climate is one of the worst for lungs in the entire country, as I am told, has demonstrated the truth of this assertion at its State consumptives' sanitarium at Rutland, where the inmates are made to take the outdoor treatment in the dead of winter when the ground is covered with snow. The same truth has been established in other cities and States where the authorities maintain open-air consumptive colonies.

But I do not wish to be understood as detracting one iota from the favorable features of the climate of Colorado and New Mexico. I merely desire to hold out hope to those who have not the means at their command to go to a climate which nature has especially adapted for consumptives. To my mind Colorado and New Mexico furnish the ideal lung climate of America. The altitude is high and air rare and invigorating. The sun fails to shine in Denver only fifty-two days out of 365 days in each year. The atmosphere even in winter is so dry that it is possible to walk on the sunny side of the street in zero weather without an overcoat. The cold is never penetrating. The fresh mountain air conduces to a good appetite. The cool nights enable one to gain splendid rest. In fact nature has assembled in those regions every element favorable to abating lung disorders. But only a comparatively small percentage of the consumptives are able to leave their homes in other parts of the country and journey to the far West, pay a high price for board, and there await the processes of nature to cure them. Their circumstances force them to remain at home, but they need not die if they will accept the open-air treatment, say right here in Alabama. A longer period will be required to abate the disease, but patience and assiduous adherence to the open-air life will in most cases, I believe, do the work, provided the beginning is made in time.

OUR DUTY IN ALABAMA.

Having considered the disease and the method if its abatement at some length I now reach the question of our duty here in Alabama. I say "our duty," for I regard it as the duty of every good citizen to aid in the solution of the problems which the subject presents, whether he be a physician or a layman. Our duty is a double one—involving both the prevention of the spread of the infection and the cure—but for convenience we will consider them jointly. It is easier to prevent any evil than to cure it. The same is true of consumption. To prevent the spread of tuberculosis we must have laws and organizations through laws and we must have enlightenment. To secure both or either the medical profession must lead the way, but it must have at its back the united support of an intelligent and sympathetic citizenship and a fearless and interested press.

In many States laws have been enacted creating anti-tuberculosis commissions. This is the case in our neighboring State of Georgia. I have recently been in communication with Dr. Bernard Wolff of Atlanta, secretary of the Georgia State Commission on Tuberculosis, and he has described to me the workings of the commission in this State. The functions of the Georgia commission are to gather data with reference to tuberculosis and to disseminate information in brief and simple form for the education of the public on precautionary methods to prevent the spread of the disease and to deal with it where it has occurred.

A short time ago Dr. Wolff, in a letter to me, said: "We are preparing to send out literature on the subject of consumption very much in the vein alluded to in your letter. We are availing ourselves of every method of disseminating this information to the public, especially through the channel represented by the newspapers. We hope in this way to arouse public sentiment in favor of the movement, and we have the narrowing conviction that once the movement is under way it will accumulate enough momentum to propel itself. I am preparing a series of tracts to be published in the newspapers. They are practical, elementary and such as any intelligent layman may understand. If the conviction can be forced into a dense and churlish public that consumption can be caught and that being caught it can often be cured, we may get rid of the old hereditary notion and the policy that have been the means of spreading the disease since the primal course. We are very much hampered by the lack of funds. The State with that easy indifference to its best interests and that cool assumption that work for it will be done free, has made no appropriation at all to carry out the work. It is maintained by assessment among the members of the commission whose efforts in the cause of cold drawn philanthropy spell professional suicide by the starvation route. With such a handicap the best we can do is to start the movement—to touch the button with the hope that if that great baby, the public, will get a good scare it will stop putting every thing into its mouth."

Dr. Wolff's views are, in my judgment, highly valuable and furnish a basis for us Alabamians to operate upon. If we are to have an anti-tuberculosis commission in this State—and we should have such a body by all means—I would suggest and urge that in addition to granting it power to collect data on the subject of tuberculosis through the medium of county and city health officers and county medical societies and issuing from time to time educational leaflets on the manner of warding off and treating the disease that it also be empowered to suggest to municipal government the enactment and enforcement of certain necessary laws providing sanitary and hygienic safeguards, such as anti-spitting ordinances, proper ventilation of public buildings, particularly the public schools, and like means for the prevention of the propagation of the tubercular germ.

SHOULD COMPILE DATA.

This commission should be supplied with a reasonable appropriation for the compilation of accurate data on the progress of tuberculosis in Alabama, and the means to be adopted to keep down the plague, for the printing and mailing of such educational tracts as it may issue from time to time, and for other necessary incidental expenses which might be legitimately incurred in its regular course of operation. The commission should invite and should have the cordial co-operation of every doctor in the State, the support of the press as a whole, and the due consideration and earnest assistance of the municipal authorities and the school trustees and boards of education in every city, town and beat in the State. The commission should hold at least two conventions yearly for the hearing of reports from its general officers and members, for the comparison of data, and for the general discussion of the subject of tuberculosis in all its practical phases.

From time to time the commission with the advice of the State Medical Association should recommend to the legislature such additional legislation as time and experience might seem to render necessary. It would in my judgment, too, be an excellent idea for the commission to agitate and press for solution the question of separate quarters at all convict farms, in all prisons and county poorhouses, for consumptive inmates. In fact, we need at this time a State sanitarium for free treatment of consumptives who are unable to secure treatment for themselves, or a sanitarium where they can be treated at nominal cost under State supervision. The region around Citronelle in the southern part of the State, I am told, is admirably adapted to such a purpose in so far as climate and natural conditions are concerned. This would be another question for the anti-tuberculosis commission to investigate and make recommendation upon.

Since the propagation of tuberculosis is brought about chiefly through poor sanitation of dwelling-houses and public buildings, due more from ignorance and carelessness than otherwise, and through the carelessness and indifference of infected persons depositing their sputum in public places, it is easy to see that a commission having in hand the quasi-regulation of these things could hope to accomplish very much. Even if it had no power to enforce its edicts it could at least educate the private citizen and the public official, and in many cases frighten both into conforming to its suggestions. However, if it accomplished nothing else, an anti-tuberculosis commission which would be able to launch a campaign of education along this line would have done lasting good. Public sentiment could be thus aroused and, as Dr. Wolff well says, "once the movement is under way it will accumulate enough momentum to propel itself."

WOULD HELP A COMMITTEE.

In conclusion, I desire to say that I am willing here and now to pledge my cordial and active support to any movement which may be inaugurated by this association, looking to the creation of a committee of intelligent and earnest men to take up this momentous question and bring it to the consideration of the next legislature and seek such legislation as will give life to such a body as I have outlined, and thus legally inaugurate a vigorous campaign in Alabama against the great "white plague." I believe that such a movement would have the united support of the press and of every thoughtful citizen of the State, and would receive the favorable consideration of our lawmakers.

I am deeply grateful for the kind attention which you have accorded me, and again wish to express to you the keen sense of appreciation which I feel in having been called upon by your honorable president to make known to you the somewhat crude but none the less sincere views of an humble layman who, having gone through the mill and emerged from it still alive though somewhat dilapidated, is enabled to comprehend and sympathize with you to the fullest extent in your great philanthropic work in behalf of suffering humanity.

---

### MASSAGE AS AN OCCUPATION FOR THE BLIND.

According to the *Medical News*, the blind have almost a monopoly of the practice of massage in Japan. In Russia, Belgium, England, and Germany, also, efforts have been made to promote the practice of this art among the blind. It would seem that the delicacy and decision of touch so often possessed in a marked degree by those deprived of the blessing of sight would render them admirably suited for the practice of massage.

